# Correlates of Not Using Antiretroviral Therapy Among Transwomen Living with HIV: The Unique Role of Personal Competence

**DOI:** 10.1089/trgh.2018.0006

**Published:** 2018-08-01

**Authors:** Richard A. Crosby, Laura F. Salazar, Brandon J. Hill

**Affiliations:** ^1^ Kinsey Institute for Research on Sex, Gender, and Reproduction, Indiana University, Bloomington, Indiana.; ^2^ College of Public Health, University of Kentucky, Lexington, Kentucky.; ^3^ Department of Health Promotion and Behavior, School of Public Health, Georgia State University, Atlanta, Georgia.; ^4^ Department of Obstetrics and Gynecology, Center for Interdisciplinary Inquiry and Innovation in Sexual and Reproductive Health, University of Chicago, Chicago, Illinois.

**Keywords:** antiretroviral therapy, counseling, HIV infections, transgender women

## Abstract

**Purpose:** This study tested three psychosocial measures for their potential to serve as counseling goals for promoting ART to transgender women living with HIV (TWLH).

**Methods:** Among 69 TWLH, 17.4% were not taking ART; these volunteers were compared to the remainder using multivariate regression analyses.

**Results:** Only one psychosocial measure achieved significance: Personal Competence (Adjusted Odds Ratio = 0.80, 95% CI = 0.67–0.97, *P* = 0.02). Because this was a continuous measure, assessed on a 7-point scale, the protective adjusted odds ratio of 0.80 represents a 20% reduction in the odds of not taking ART for each unit of increase in this construct.

**Conclusion:** Findings suggest a potential counseling goal for TWLH not taking ART.

## Introduction

Although advances in the treatment of HIV/AIDS have been revolutionary, the full potential of these chemotherapeutic agents to reverse the HIV epidemic has yet to be realized. Latest estimates, for instance, are that only about 25% of those living with HIV are virally suppressed.^1^ A majority of those not taking antiretroviral therapy (ART) are not engaged in medical care.^2–4^ Estimates suggest that upwards of 50% of people diagnosed with HIV/AIDS are not in continuous care.^5^ Furthermore, estimates suggest that 25% of persons who are engaged in care and eligible for ART are not receiving this therapy.^5^ The public health issue of people living with HIV and not taking ART is one that has been aggressively addressed with empirical studies.^6,7^ A key population, however, that has been largely overlooked in efforts to better understand the nonuse of ART is transwomen. Transgender women experience a high burden of HIV worldwide with pooled prevalence estimates at 19.1% (95% confidence interval [CI] 17.4–20.7).^1^ In the United States, the burden of HIV for transgender women may be even higher with average prevalence rates among the highest of all risk groups.^8–10^

Because transwomen living with HIV (TWLH) can be a very difficult population to access for research studies, the few extant studies of TWLH are limited in sample size and thus may have been lacking in adequate power. Nonetheless, these studies remain valuable for their ability to inform intervention efforts. For instance, a study of 35 TWLH assessed multiple correlates of nonadherence to ART, for example, depression, adherence self-efficacy, patient–provider interactions, and perceived side effects.^11^ A study of 59 TWLH also assessed correlates of nonadherence, for example, age, stress, transphobic experiences, and hormone replacement therapy (HRT).^12^ A study of 113 TWLH found that recent psychosocial trauma was associated with nonadherence to ART.^13^

Given that nationally representative data suggest greater proportions of TWLH not using ART than cisgender males living with HIV,^14^ it is important to have a continued research focus designed to identify correlates of this nonuse in TWLH. In particular, correlates that may be amenable to change through counseling have the opportunity to inform practice. Indeed, a primary research question that remains unexplored in the published literature involves targeted counseling goals for transgender women living with HIV, but not taking ART. Counseling goals, by definition, must be amenable to change at the individual-level.

Two general counseling goals for transwomen often involve building resilience and improving satisfaction with body image.^15^ Resilience is best conceptualized as the primary protective factors against stress and various forms of trauma.^16,17^ A recent study found evidence suggesting that two forms of resilience (personal competence, and acceptance of self and life) may be important outcomes of socially conferred gender affirmation.^18^ Among transwomen, resilience may also be an outgrowth of a favorable body image.^19^ A favorable body image, in turn, may be a robust protective factor for transwomen, independently from resilience.^20^ Objectification theory provides a strong set of tenets as to why body image is so often a vital aspect of the self among transwomen.^21,22^

Accordingly, the purpose of the study was to test three psychosocial measures for their potential to serve as counseling goals in service of the larger goal of using ART. The three measures were as follows: (1) personal competence, (2) acceptance of self and life, and (3) body image. We hypothesized that each of these three constructs would be independently associated with the outcome of ART use, after controlling for variables that are not readily amenable to serve as counseling goals. Specifically, we hypothesized that TWLH having more favorable levels of each measure would be more likely to adhere to ART use. In recognition that these three measures would not be independent, their respective influence was assessed using multivariate regression.

## Methods

### Participants and procedures

Data were taken from a larger study (*N*=161) of both HIV-infected (*N*=69) and HIV-uninfected (*N*=92). Multiple community-based outreach strategies were used to recruit this community sample of transwomen between ages 18 and 65 years, residing in Atlanta, Georgia, or Chicago, Illinois. Venues serving transwomen as well as word-of-mouth from transgender advocates served as the primary methods for recruitment. In addition, the project was advertised through formal and informal communication channels via advocacy groups and lesbian, gay, bisexual, and transgender (LGBT) service organizations. Also, recruiters approached transwomen in the community and provided information about the study. Word-of-mouth and flyers placed in community venues serving transgender women were also used. Print materials provided contact information for the project coordinator. Data were collected from October 2014 through June 2015 in Atlanta and from April through October of 2017, in Chicago.

All participants were screened to determine eligibility. Requirements included the following: (1) being 18 to 65 years of age; (2) having been assigned male at birth and self-identifying as either a transgender woman, female, or gender-nonconforming identity; and (3) reporting anal sex with a cisgender male or nontransgender male in the past 6 months. After providing written informed consent, participants were engaged in a face-to-face structured interview with a trained graduate research assistant lasting ∼60–90 min. All interview survey responses were recorded on a portable electronic tablet using Qualtrics© software (Provo, UT). Self-report of HIV status as “positive” was used to classify the analytic subset for this study (*N*=69). The Institutional Review Boards for research on human subjects at Georgia State University and the University of Chicago approved all study protocols and procedures.

### Measures

For the outcome measure, a single-item assessed whether TWLH were currently taking ART. For possible control variables, in addition to standard sociodemographic characteristics, we assessed several factors: (1) current use of estrogen, (2) whether participants had a legal name change to reflect their gender identity, (3) whether participants were currently involved in what they considered to be a committed relationship, (4) whether participants had health insurance (including Medicaid as an option) at the time of study enrollment, and (5) an ordinal measure of placement in terms of transitioning to a woman. This latter measure was simply devised by our study team to reflect various degrees of the transition process (not started, recently started, somewhat transitioned, mostly transitioned, and completely transitioned).

For the predictor variables, three scale measures were assessed as the factors of interest for this study given our intent to identify potential counseling-amenable targets for change that may best foster ART use among those not taking advantage of this innovation in HIV/AIDS care.

#### Personal competence

As a subscale of the Wagnild and Young Resilience Scale,^22^ a six-item measure was used to represent personal competence in managing daily life (Table 1). Response options included a seven-point Likert scale, 1=strongly disagree, 7=strongly agree. Interitem reliability was excellent, with a Cronbach's alpha=0.86.

**Table 1.** **Items Comprising the Two Subscales Derived from the Wagnild & Young Measure of Resilience**

Personal competence
I usually manage one way or another
I feel proud I have accomplished things in life
I am friends with myself
I have self-discipline
I can usually find something to laugh about
My life has meaning
Acceptance of self and life
Keeping interested in things is important to me
I usually take things in stride
I keep interested in things
I can usually look at a situation in a number of different ways
I am able to depend on myself more than anyone
I take things one day at a time

#### Acceptance of self and life

Also, a subscale of the Wagnild and Young Resilience Scale,^22^ a six-item measure was used to represent managing stress through positive and proactive (Table 1). Response options included a seven-point Likert scale (as above). Interitem reliability was excellent, with a Cronbach's alpha=0.88.

#### Body image

A seven-item scale (created by the authors) assessed body image. Interitem reliability was excellent, with a Cronbach's alpha=0.83. Items were prefaced with the question stem, “How happy are you with…” and seven different words followed: face, arms, chest, legs, feet, hair, and voice. Response options were based on a five-point scale ranging from 1 (very happy) to 5 (very unhappy).

### Data analysis

First, bivariate associations between each possible control variable (Table 2) and the outcome of using ART were assessed through either the use of chi-square or *t*-tests, depending on the metric of measurement. Using a screening value of *p*<0.20, control variables were then selected for use in a hierarchal logistic regression model. This model was constructed specifically to test the study hypothesis, thus the three scale measures were entered in a second block with the first block being used only for direct entry of the selected control variables. Significance for the model was defined by a *p-*value of <0.05.

**Table 2.** **Observed Bivariate Associations Between Possible Control Variables and Nonuse of Antiretroviral Therapy Among Transgender Women Living with HIV**

Correlates tested by Chi-square	% Not taking ART	Chi-square value	*p*
City of recruitment			
Atlanta (*n*=50)	16.0	0.24	0.62
Chicago (*n*=19)	21.1		
Annual net income <$10,000			
Yes (*n*=45)	17.8	0.01	0.91
No (*n*=24)	16.7		
Current use of estrogen			
Yes (*n*=33)	6.1	5.65	0.017
No (*n*=36)	21.1		
Currently have health insurance			
Yes (*n*=41)	12.2	0.90	0.17
No (*n*=28)	25.0		
Had legal name change			
Yes (*n*=33)	12.1	1.22	0.27
No (*n*=36)	22.2		
Currently in a meaningful relationship			
Yes (*n*=29)	10.3	1.73	0.19
No (*n*=40)	22.5		

Correlates tested by *t*-tests	Mean if not taking ART	Mean if taking ART	*p*
Age in years	26.9	36.5	0.005
Transition status^a^	2.50	3.45	0.018
Personal Competence Scale^b^	33.4	38.5	0.002
Acceptance of Self and Life Scale^c^	34.5	38.1	0.03
Body Image Scale^d^	23.5	28.2	0.009

^a^ This was assessed on a five-point scale ranging from 1=not begun to 5=completed.

^b^ Scores ranged from 4 to 42, with higher scores representing greater personal competence.

^c^ Scores ranged from 6 to 42, with higher scores representing greater acceptance of self/life.

^d^ Scores ranged from 7 to 35, with higher scores representing greater satisfaction w/body image.

ART, antiretroviral therapy.

## Results

### Characteristics of the sample

The sample of 69 transgender women ranged from 19 to 65 years of age (mean=34.9; standard deviation [SD]=10.9). Most (95.7%) identified as black or African American. Slightly less than one-half of the sample (44.9%) indicated receiving education beyond high school. Current employment was indicated by 30.4%. About two-thirds of the sample (65.2%) reported an annual income (after taxes) of less than $10,000. Five (7.2%) reported having sex with one or more biological females in the past 6 months. The average number of biological male sex partners in the past 6 months was 3.25 (SD=6.55), with a range of 0 to 50 and a median of 1.0. During this same 6-month recall period, the mean number of male partners who engaged in anal sex with participants was 3.05 (SD=6.76), with a range of 0 to 50. The mean number of male partners who engaged in oral sex with participants was 2.65 (SD=4.30), with a range of 0 to 25. About one-sixth of the sample (15.4%) indicated that one or more of the males they had sex with in the past 6 months was a partner who gave money or drugs in exchange for sex. Of the 69 TWLH, 17.4% were not taking ART.

### Bivariate associations

Table 2 displays the observed bivariate associations. As shown, three dichotomous correlates (health insurance, use of estrogen, and a current meaningful relationship) achieved the screening significance level of <0.20. Also as shown, age, transition status, and each of the three scale measures were well under the screening level of bivariate significance.

Finally, we determined the correlations between the three psychosocial measures. The measure of body image was significantly correlated with the measure of acceptance of self and life (*r*=0.42). Furthermore, body image was significantly correlated with the measure of personal competence (*r*=0.45). Also, the measure of acceptance of self and life was significantly correlated with perceived competence (*r*=0.87).

### Multivariate associations

Table 3 displays the adjusted odds ratios plus their 95% CIs and *p* values relative to the variables retained in the logistic regression model. As shown, only one achieved multivariate significance: Personal Competence. Because this was a continuous measure, assessed on a seven-point scale, the protective adjusted odds ratio of 0.80 represents as 20% reduction in the odds of not taking ART for each unit of increase in this construct.

**Table 3.** **Adjusted Odds Ratios, 95% Confidence Intervals, and *p*-Values of Variables Retained in the Logistic Regression Model**

Correlate	Adjusted odds ratio	95% CI	*p*
Age	0.96	0.86–1.07	0.42
Currently not taking estrogen	1.52	0.20–11.55	0.68
Currently have health insurance	0.14	0.02–1.06	0.06
Transition status	0.55	0.24–1.25	0.16
Currently not in a meaningful relationship	0.57	0.08–3.97	0.58
Personal competence	0.80	0.67–0.97	0.02

CI, confidence interval.

## Discussion

In an era of treatment as prevention being the dominant approach to the control of HIV, this was an important initial investigation pertaining to a highly marginalized population, TWLH. Findings support the strong association of a construct labeled personal competence as a counseling goal for TWLH who are not currently using ART. Specifically, counseling goals related to helping TWLH gain an increased sense of managing life, instilling self-discipline, finding meaning in life and pride in their accomplishments, and having a general sense of self-liking may all be valuable goals in terms of promoting their long-term use of ART.

Notably, the vast majority of the sample identified as black/African American. Fostering this type of resilience may be especially important for this population of TWLH given the historical significance of structural and institutional racism that likely intersects with poverty, sexism, and transphobia experienced by black transgender women in the United States.^23,24^ Furthermore, the bivariate significance of the other two scales tested (acceptance of self and life, and body image) suggests that these factors may also be important in other populations of TWLH. Whether, for instance, either of these scales would be significant in a multivariate model of a sample, including a lesser proportion of black/African American TWLH is worthy of future investigations.

In addition to the counseling implications, several other practice-based implications are suggested by the study findings. For example, current use of exogenous estrogen was associated with a threefold decrease in the odds of not taking ART. This observation raises the possibility that providers of HRT to TWLH may be, in parallel, promoting adherence of ART. Clearly, HRT may be a strong driver for seeking health services among transgender women, thus linking this service to ART promotion/access is an important public health practice. As a second example, the strong bivariate association between transition status and ART use suggests that TWLH who are *less advanced* in the transitioning process may be in greater need of intensified counseling and outreach efforts designed to maintain the uptake of, and adherence to, ART. Finally, given the small sample size, the near (i.e., *p*=0.06) multivariate significance of having health insurance is important as it underscores the value of Medicaid and other “safety net” insurance programs for promoting ART use and thus serving a primary goal of public health in the era of HIV/AIDS.

Study findings provide a modest, yet novel, contribution to a growing body of empirical evidence relative to HIV prevention and treatment for transgendered women.^24–28^ This implication that a focus on building personal competence may be a beneficial counseling goal for transgender women coincides with rapidly growing attention and investment in the HIV/AIDS prevention, care, and treatment of this population. The challenge, of course, in applying this implication to practice is that counseling can only occur for transgender women connected to care. We suggest, however, that a “middle-ground” may exist in that posttest HIV counseling, when HIV testing is done in nonclinical settings, could be an opportune time to at least initiate a counseling relationship with transgender women testing reactive. Given a recent estimate from CDC that more than 60% of U.S. transgender women have not been tested for HIV,^27^ coupling community-based HIV testing/counseling for those testing positive with an interim avenue for ongoing counseling may help promote their eventual uptake of, and adherence to, ART.

### Limitations

Many of the study participants were recruited through referrals from community-based organizations providing services and support to transgender women. Whether these women are representative of transgender women not receiving similar support and services is not known. Furthermore, the majority of our sample identified as non-Hispanic black, thus the findings may not generalize to other racial/ethnic populations of TWLH. Also, that the two subscales derived from the larger 25-item measure of resilience were constructed and used in the same sample is a potential limitation. Finally, the small sample size and corresponding lack of adequate statistical power may have masked otherwise significant and important study findings. We clearly acknowledge that having only 12 of the 69 transwomen report not taking ART is a study limitation. Had a larger proportion of the study participants reported not taking ART, the available statistical power would have increased thereby potentially giving two variables a greater influence: having health insurance and a current meaningful relationship.

## Conclusion

This is one of the relatively larger community-based studies of TWLH, a difficult to access population. The findings suggest a potential counseling goal for TWLH not taking ART. Community-based counseling for transgender women recently testing positive for HIV may benefit this population by seeking to help them construct more favorable perceptions of their personal competence. This goal may be achieved by providing individualized counseling, tailored to meet the needs of TWLH relative to the acquisition of enhanced personal competence.

## Abbreviations Used

ART: antiretroviral therapy
CI: confidence interval
HRT: replacement therapy
SD: standard deviation
TWLH: transwomen living with HIV
